# Multi-Level Cycle-Consistent Adversarial Networks with Attention Mechanism for Face Sketch-Photo Synthesis

**DOI:** 10.3390/s22186725

**Published:** 2022-09-06

**Authors:** Danping Ren, Jiajun Yang, Zhongcheng Wei

**Affiliations:** 1 Hebei Key Laboratory of Security Protection Information Sensing and Processing, Handan 056038, China; 2 School of Information and Electrical Engineering, Hebei University of Engineering, Handan 056038, China

**Keywords:** image transformation, face sketch-photo synthesis, convolutional block attention module, generative adversarial network, multiscale feature

## Abstract

The synthesis between face sketches and face photos has important application values in law enforcement and digital entertainment. In cases of a lack of paired sketch-photo data, this paper proposes an unsupervised model to solve the problems of missing key facial details and a lack of realism in the synthesized images of existing methods. The model is built on the CycleGAN architecture. To retain more semantic information in the target domain, a multi-scale feature extraction module is inserted before the generator. In addition, the convolutional block attention module is introduced into the generator to enhance the ability of the model to extract important feature information. Via CBAM, the model improves the quality of the converted image and reduces the artifacts caused by image background interference. Next, in order to preserve more identity information in the generated photo, this paper constructs the multi-level cycle consistency loss function. Qualitative experiments on CUFS and CUFSF public datasets show that the facial details and edge structures synthesized by our model are clearer and more realistic. Meanwhile the performance indexes of structural similarity and peak signal-to-noise ratio in quantitative experiments are also significantly improved compared with other methods.

## 1. Introduction

Face sketches and face photos can be converted into one another. As the technology of face photo-to-sketch synthesis becomes more mature, it is widely used in digital entertainment, public security law enforcement, and case investigation [1]. For example, the suspect’s face photos taken by surveillance cameras often have the conditions of occlusion and low resolution, which affect face recognition. Law enforcement agencies have to ask artists to draw face sketches of suspects based on eyewitness accounts and surveillance videos. However, there is a large modal gap between face photos and face sketches, so it is difficult to achieve accurate recognition. Therefore, it can solve the above problems quickly by converting face sketches to face photos. Meanwhile, sketches are more artistic than photos in digital entertainment, as more users upload their sketch portraits to social platforms.

The traditional exemplar-based methods divide the image into overlapping patches and operate at the patch level. These exemplar-based methods synthesize target images by matching and combining image patches. However, exemplar-based methods often have the disadvantages of being time-consuming, requiring a large amount of data, and generating sketches that are too smooth. With the rapid development of deep learning, many sketch synthesis methods based on convolutional neural networks (CNN) have emerged. A benefit from the adversarial loss is that the sketches synthesized by the GAN-based methods are more realistic. However, due to the lack of special constraints, the generated images also have blurring artifacts. There has been a lot of work on face photo-to-sketch synthesis, but less research work on face sketch-to-photo synthesis. Although some methods to solve the former can be used in the latter, the sketch-to-photo synthesis is a process of information ascent leading to issues, such as a lack of detail and blurred edges in the synthesized facial photos. At the same time, due to the limited amount of pairing sketch-photo data nowadays, the collection and production would consume a lot of energy and resources. Therefore, this paper proposes an unsupervised generative adversarial network to achieve a higher-quality face sketch-to-photo synthesis.

First of all, this paper adopts the basic network structure of CycleGAN [2]. Previous GAN-based synthesis methods normally only use a single-scale convolution kernel for feature extraction. Sketch images have different styles and texture information features at different scales, such as large-scale line features and small-scale shadow features. The previous single-scale feature extraction cannot meet the needs of sketch-to-photo synthesis. Considering the rich texture structure in face sketches, this paper adopts different styles and scales of convolution and pooling ways to form a multi-scale feature extraction module. It can extract feature information of multiple scales to add the multi-scale feature extraction module (MFEM). The benefit from MFEM include that the reconstructed face photo retains more semantic information that is similar to the face sketch. In the process of converting sketches to photos, the reconstruction of the facial area is obviously more important than the reconstruction of the background area, therefore, we should strengthen the constraints on facial features. The Convolutional Block Attention Module (CBAM) [3] is introduced into the residual block of the generator network, thus enhancing the representation ability of the network structure, making the model focus on more important feature information and suppressing unnecessary characteristic information. Secondly, compared with other unsupervised image translation models, this paper not only applies the pixel-level cycle consistency loss, but also increases the perceptual loss and facial detail feature loss proposed from a global and regional perspective. The multi-level cycle consistency loss composed of the three is applied in the model, which greatly reduces the information loss during the conversion process and retains more facial structure information. Compared to six existing models on CUFS and CUFSF dataset, the experimental results of the proposed model show better qualitative and quantitative performance.

In summary, the main contributions of our paper are as follows:

- Considering that sketches contain texture feature information of different scales, we add MFEM before the generator, enabling the network to extract multi-scale feature information.
- We add CBAM to the residual block of the generator to improve the ability of the model to extract important feature information for better synthetic results.
- Based on the CycleGAN method, we construct multi-level cycle consistency loss to preserve the key facial features. Experimental results show that the photos synthesized by our method are more real and clear.

## 2. Related Work

### 2.1. Face Photo—Sketch Synthesis 

Face photo-sketch synthesis can be traced back to the traditional synthesis method of the image patch level. These include local linear embedding methods based on subspace learning [4], Markov random field models based on Bayesian theory [5], and Markov weight field models [6], as well as methods based on sparse representation [7]. However, the face image synthesized by traditional methods has a fuzzy effect, which leads to the lack of real face details being to too smooth. In recent years, with the rapid development of deep learning, many face sketch synthesis methods based on convolutional neural network have been proposed. Zhang et al. [8] can obtain a rough face sketch by constructing a branch-full convolution network. However, the sketch synthesized by the model is not able to retain more facial details. As generative adversarial networks (GAN) [9] show more powerful generative capabilities, it is also widely used in the field of image translation. Wang et al. [10] supervised the hidden layer of the generator through multiple discriminators and iterated the low resolution image into a high-resolution image, which solved the problem of low resolution of the synthetic image, to a certain extent. Fang et al. [11] proposed a new identity awareness cycle generation countermeasure network model. The model combines the synthesis model and the recognition model to optimize each other, which not only improves the image quality, but also improves the accuracy of recognition. Chao et al. [12] added the residual block to u-net as a new generator and designed an effective loss function to enhance the pixels, edges, and high-level features of the generated face photos. This model effectively generates high fidelity images. Zhu et al. [13] proposed a collaborative framework for mapping sketches and photos to each other, which was set to map them to the same potential domain in order to retain more common information between the two domains. Yu et al. [14] proposed a framework for generating confrontation networks combined with facial prior information. This model uses the decomposed single label of facial pixels to help synthesize the target domain image, and solves the fuzzy deformation problem of facial components. Isgan [15] ensures that the synthetic image retains more recognizable information by embedding identity information in the training process and using new network losses. Although the above methods improve the quality of synthesized images, they have the limitation of requiring paired sketch-photo data for training.

### 2.2. Attention Mechanism 

In recent years, attention models have been widely used in various deep learning tasks such as image recognition, image super-resolution, image translation, and image classification. Adding an attention mechanism to a convolutional neural network can expand the expressiveness of the model and achieve better results. Bahdanau et al. [16] introduced an attention mechanism to significantly improve the translation performance of machine translation models. Hu et al. [17] adaptively adjusted the channel feature information by stacking Squeeze-and-Excitation blocks. Woo et al. built a spatial attention module based on SENET and integrated spatial and channel information to obtain more comprehensive attention information. Fu et al. [18] combined FCN with CBAM and proposed a DANet to build dependencies between local features and global features to improve the segmentation performance of the model. Zhang et al. [19] added a self-attention mechanism to GAN and proposed SAGAN to improve the ability of the model to capture long-range dependencies.

## 3. Proposed Method

### 3.1. Framework

**Figure 1:**
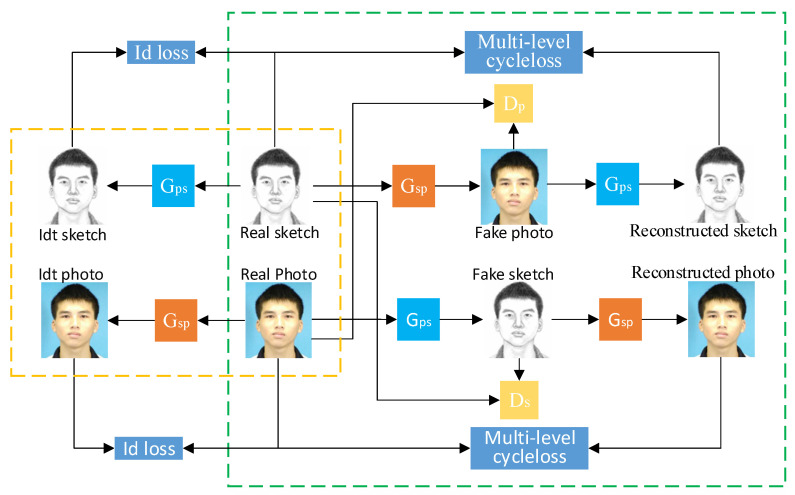
Overall network framework.

The goal of our model is to transform the face sketch into the face photo without supervision (without pairing data). Given the samples of unpaired face sketches *S* = {*S_i_*, *i* = 1, 2, 3,…, *n*} and face photos *P* = {*P_i_*, *i* = 1, 2, 3,…, *n*}, the overall framework structure is shown in Figure 1, which includes two generators *G_sp_* and *G_ps_* and two discriminators: *D_s_* and *D_p_*. The model proposed in this paper mainly studies the mapping relationship between the face sketch and the face photo. Real face sketches, *Real_s,* are converted to the synthetic face photos, *Fake_p,* by the generator, *G_sp_*. Synthetic face, *Fake_p,* can also regenerate reconstructed sketches, *Cyc_s,* through *G_ps_*. The network branch above the right frame of Figure 1 can be expressed as Formula (1):

(1) $$Fake\_p=G_{sp}\left(Real\_s\right),Cyc\_s=G_{ps}\left(Fake\_p\right)$$

Similarly, the network branch under the right box can be expressed as Equation (2):

(2) $$Fake\_s=G_{ps}\left(Real\_p\right),Cyc\_p=G_{sp}\left(Fake\_s\right)$$

The discriminator model in this paper adopts PatchGAN [20], which divides the whole image into areas for judgement. The purpose of the discriminator *D_s_* is to distinguish the unpaired real face sketches, *Real_s,* and the synthetic face sketches, *Fake_s*. Similarly, the purpose of the discriminator, *D_p_*, is to distinguish the unpaired real face photos, *Real_p*, and the synthetic face photos, *Fake_p*. The results fed back to the generator by *D_s_* and *D_p_* are used to iteratively optimize the whole model. The process of the model is shown in Figure 1. After extracting multi-scale features from the input sketch, the corresponding photos are synthesized by the generator, *G_sp_*. At the same time, CBAM is used to adjust the weight of important information during the conversion process. The synthesized photos are reconstructed into sketches through the generator *G_ps_*. The multi-level cycle consistency loss is used to supervise the generation of synthesized photos with more details preserved. Finally, the discriminator, *D_p_*, is used to distinguish real photos and synthesized photos, and gradually reduce the distance between them.

### 3.2. Network Structure

**Figure 2:**
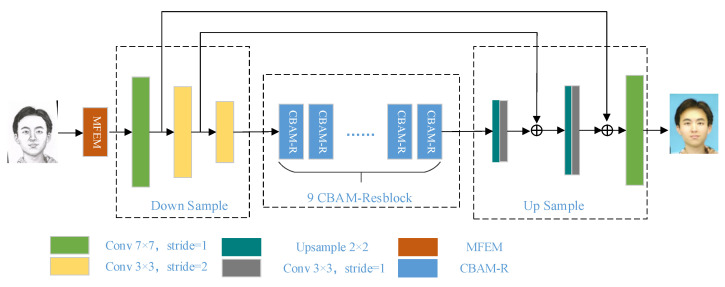
Generator network structure.

The overall network architecture for converting face sketches into face photos is illustrated in Figure 2. In order to make the synthesized target photo domain image retain more abundant semantic information, a multi-scale feature extraction module is added in front of the generator. Meanwhile, the attention mechanism is introduced into the residual block to form a new convolution attention residual block, so that the network can better capture the important feature information and ignore the redundant information such as background.

#### 3.2.1. Generator Network Structure

In Generative Adversarial Networks, the deeper generator network can process more location information and feature information to ensure that the generated photos are more realistic and of higher quality. However, the superposition of network layers often causes problems such as gradient dispersion, which leads to the failure of the network to converge. Drawing on the deep Residual Network (ResNet) [21], the generator in this paper uses residual blocks with shortcut connections as components to avoid gradient dispersion. Although the stacked residual blocks reduce the difficulty of training and extract rich feature information, the process of down-sampling will inevitably lead to the loss of image feature information. Therefore, skip connections are added after the down-sampling and up-sampling convolutional layers to reduce the information loss during image transformation.

The network structure of the generator is shown in Figure 2. The encoding part contains three convolutional layers. The size of the convolution kernel of the first layer is 7 × 7, and the size of the other two layers is 3 × 3. The middle part consists of 9 convolutional attention residual blocks combined with attention mechanism. Finally, the decoding part is composed of two deconvolution layers and one convolutional layer. In the decoding part, the up-convolutional layers often use traditional transposed convolutions. However, the transposed convolution operation usually causes the generated image to have a checkerboard effect. Therefore, this paper uses the method of combining up-sampling and convolution to replace the transposed convolution. At the same time, Instance Normalization (IN) and ReLU activation function operations are performed on the feature map after each convolution operation.

#### 3.2.2. Multi-Scale Feature Extraction Module

Inputting the source sketch domain directly into the generator will cause the output target photo domain to have difficultly retaining facial details due to insufficient extracted feature information. In order to adapt to the rich line textures of the sketch domain, the feature information of different scales in the source sketch domain should be extracted in a multi-scale way. Therefore, the constructed Multi-scale Feature Extraction Module (MFEM) is added before the generator to ensure that the generated face photos retain more rich semantic information as the input sketch face image.

**Figure 3:**
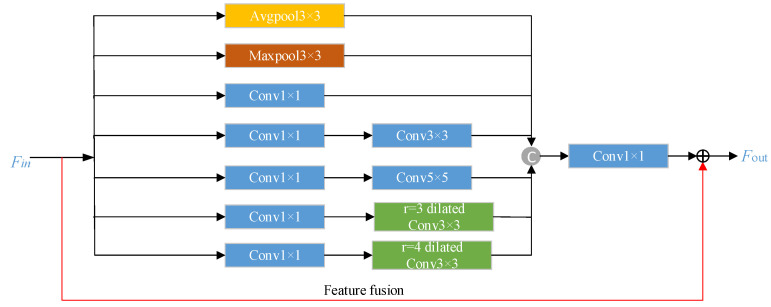
Multiscale feature extraction module.

The module adopts different ways and different sizes of convolution and pooling operations to extract multi-scale feature information. As shown in Figure 3, borrowing from the Inception basic network [22], the module as a whole is composed of two pooling branches and five convolution branches. The pooling branches include two methods: average pooling and maximum pooling. In order to obtain a larger receptive field range with fewer parameters, the dilated convolution branches with dilatation rates of 3 and 4 are added to the convolution branches of the Inception network. The receptive fields of each convolution branch are 1 × 1, 3 × 3, 5 × 5, 7 × 7, and 9 × 9, respectively. Instance normalization (IN) and ReLU activation function operation is performed after each convolution operation. Finally, the multi-scale features obtained by each line are concatenated and fused at the channel level. To avoid gradient vanishing, we used identity mapping to sum the input features and multi-scale features.

#### 3.2.3. Convolution Attention Residual Block

**Figure 4:**
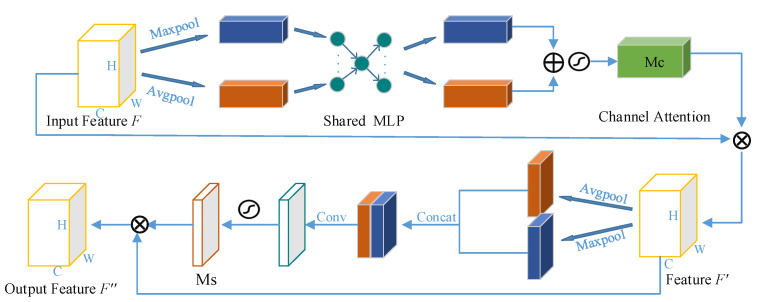
Convolutional block attention module.

It is well known that attention plays an extremely important role in human perception. Similar to the processing mechanism of the human visual system, the visual attention mechanism is also designed to highlight certain significant features. CBAM considers the difference of content information and location information in the input feature map from the two dimensions of channel and space. As shown in Figure 4, the above branch shows the operation process of the feature map by the channel attention module. First, two one-dimensional feature vectors are obtained by applying two pooling methods to the input feature map *F*, which are sent to the multi-layer perceptron (MLP) network with one hidden layer and activated through the sigmoid function to generate a channel attention map *M_c_* ∈ *R^C^*^×1^^×1^. Finally, it can be expressed by Equation (3):

(3) $$\begin{array}{ll} M_{c}\left(F\right) & =\sigma \left(MLP\left(AvgPool\left(F\right)\right)+MLP\left(MaxPool\left(F\right)\right)\right)\\ & =\sigma \left(W_{1}\left(W_{0}\left(F_{avg}^{c}\right)\right)+W_{1}\left(W_{0}\left(F_{max}^{c}\right)\right)\right) \end{array}$$

The lower branch of Figure 4 shows the operation process of the feature map by the spatial attention module. Compared with attention in channel dimension, attention in spatial dimension pays more attention to the location of feature information. After obtaining the feature map, *F*′ with the channel attention weight, the channel information is aggregated along its channel axis using two methods: max pooling and average pooling. After concatenating and convolving the two pooled feature vectors, a spatial attention map, *M_s_* is generated, which can be expressed by Equation (4):

(4) $$\begin{array}{ll} M_{s}\left(F^{\prime }\right) & =\sigma \left(f^{7\times 7}\left(\left[AvgPool\left(F^{\prime }\right),MaxPool\left(F^{\prime }\right)\right]\right)\right)\\ & =\sigma \left(f^{7\times 7}\left(\left[{F^{\prime }}_{avg}^{};{F^{\prime }}_{max}^{}\right]\right)\right) \end{array}$$

To sum up, after inputting the feature map *F*, it passes through the attention modules in the two dimensions of channel and space in turn. After the feature map is multiplied by the attention weight map, the feature information is adaptively adjusted to obtain a new feature map *F*″. Thus, the overall process can be described as follows:

(5) $$F^{\prime }=M_{c}\left(F\right)⊗F$$

(6) $$F^{\prime \prime }=M_{s}\left(F^{\prime }\right)⊗F^{\prime }$$

**Figure 5:**
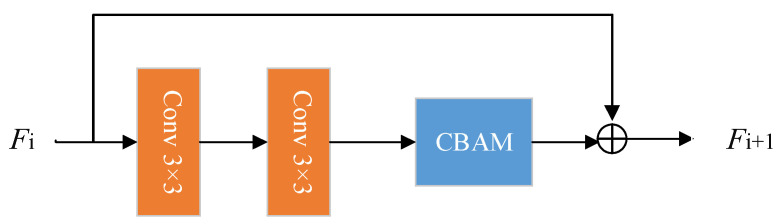
Convolutional attention residual block.

As shown in Figure 5, the CBAM is added to the residual block in the generator to form a convolutional attention residual block. The convolutional attention residual block includes two convolutional layers with convolution kernels of size 3 × 3 and a convolutional attention unit. Finally, a skip connection is added between the original input and the feature map obtained by convolution. The original convolution operation extracts features without distinguishing channel information and spatial information, and the residual part will bring redundant information that is not conducive to generate high-quality photos. The network that joins the CBMA can extract more important detailed features and improve the expressive ability of the network by applying the attention mechanism of the two dimensions of channel and space in sequence.

### 3.3. Loss Function

The loss function consists of three parts, namely adversarial loss, identity consistency loss, and multi-level cycle consistency loss. Adversarial loss constraints generate images closer to real photos. The identity consistency loss ensures that the mapping relationship is more accurate. The multi-level cycle consistency loss confirms the generated image retains more facial details, while ensuring the stable training process.

#### 3.3.1. Adversarial Loss

To avoid gradient dispersion and to update the generator more smoothly, this paper adopts the least-squares loss function [23] as the adversarial loss. It has the advantage of penalizing erroneous samples that are judged to be correct but are further away from the judgement boundary, allowing these erroneous samples to continue to be optimized iteratively. For the mapping relationship *G_sp_*: *S*→*P* and its discriminator, *D_p_*, the adversarial loss using the least squares loss function is expressed as Equation (7):

(7) $$L_{LSGAN_{p}}\left(D_{p},G_{sp},s_{i},p_{i}\right)=E_{p_{i}\sim P_{data}\left(p_{i}\right)}\left[{\left(D_{p}\left(p_{i}\right)\right)}^{2}\right]+E_{s_{i}\sim P_{data}\left(s_{i}\right)}\left[{\left(D_{p}\left(G_{sp}\left(s_{i}\right)\right)-1\right)}^{2}\right]$$

where *p_i_*~*P_data_*(*p_i_*) is the probability distribution obeyed by the optical photo sample *P*, and *s_i_*~*P_data_*(*s_i_*) refers to the probability distribution obeyed by the sketch image sample *S*. The goal of the generator, *G_sp_*, is to minimize the objective function to make the synthesized optical photo image closer to the real photo sample. The goal of the discriminator, *D_p_*, is to maximize the objective function to correctly distinguish the generated optical photo images from the real optical photo samples. Similarly, for mapping *G_ps_*: *P*→*S* and its discriminator, *D_s_*, the adversary loss target using the least square loss function is expressed as Equation (8):

(8) $$L_{LSGAN_{s}}\left(D_{s},G_{ps},s_{i},p_{i}\right)=E_{s_{i}\sim P_{data}\left(s_{i}\right)}\left[{\left(D_{s}\left(s_{i}\right)\right)}^{2}\right]+E_{p_{i}\sim P_{data}\left(p_{i}\right)}\left[{\left(D_{s}\left(G_{ps}\left(p_{i}\right)\right)-1\right)}^{2}\right]$$

#### 3.3.2. Identity Consistency Loss

The mapping relationship between the generators *G_sp_* and *G_ps_* is *S*→*P* and *P*→*S*, respectively. To make the mapping relationship of the generator more accurate, we follow CycleGAN to generate the same samples after inputting the target domain samples into the generator. To this end, an identity consistency loss is constructed between the input image and the generated image. Experiments showed that adding an identity consistency loss could make the tones of the generated images closer to the real samples. The identity consistency loss is expressed as Equation (9):

(9) $$L_{Identity}\left(G_{sp},G_{ps}\right)=E_{s_{i}\sim P_{data}\left(s_{i}\right)}\left[G_{ps}\left(s_{i}\right)-s_{i}\right]+E_{p_{i}\sim P_{data}\left(p_{i}\right)}\left[G_{sp}\left(p_{i}\right)-p_{i}\right]$$

#### 3.3.3. Multi-Level Cycle Consistency Loss

CycleGAN constructs a cycle consistency loss according to the mapping relationship between the source domain and the target domain, which constrains the sketch domain image to be converted into the sketch domain image after synthesizing the photo domain image. The mapping relationship is as follows: *s_i_*→*G_sp_*(*s_i_*)→*G_ps_*(*G_sp_*(*s_i_*)) ≈ *s_i_*, *p_i_*→*G_ps_*(*p_i_*)→*G_sp_*(*G_ps_*(*p_i_*)) ≈ *p_i_*. In CycleGAN, the pixel-level cycle consistency loss is adopted, As Equation (10):

(10) $$L_{cyc\_pix}\left(G_{sp},G_{ps}\right)=E_{s_{i}\sim P_{data}\left(s_{i}\right)}\left[G_{ps}\left(G_{sp}\left(s_{i}\right)\right)-s_{i}\right]+E_{p_{i}\sim P_{data}\left(p_{i}\right)}\left[G_{sp}\left(G_{ps}\left(p_{i}\right)\right)-p_{i}\right]$$

**Figure 6:**
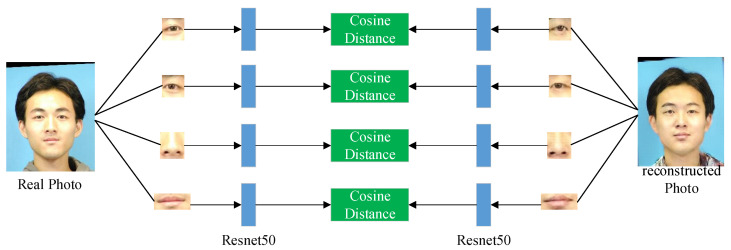
Loss calculations with facial detail retained.

For image synthesis problems with large modal differences between two domains, the model only adopts pixel-level cycle consistency loss and often fails to learn high-level features of source domain images, which affects the training process. Borrowing from Johnson [24], an additional perceptual loss is adopted to constrain the Euclidean distance between high-level features. We use the VGG-19 network trained on the ImageNet dataset to extract the features of the source domain image and the reconstructed image, and then the two are compared to preserve more detailed textures. The five layers of ReLU1_1, ReLU2_1, ReLU3_1, ReLU4_1, and ReLU5_1 in the VGG-19 network are used as the feature output part. Therefore, the cycle consistency loss at the global feature level is adopted, as shown in Equation (11):

(11) $$L_{cyc\_feature}\left(G_{sp},G_{ps}\right)=\sum_{j=1}^{5}\left(\frac{1}{N_{j}}{\left\|\Phi_{j}\left(s_{i}\right)-\Phi_{j}\left(G_{ps}\left(G_{sp}\left(s_{i}\right)\right)\right)\right\|}_{2}^{2}+\frac{1}{N_{j}}{\left\|\Phi_{j}\left(p_{i}\right)-\Phi_{j}\left(G_{sp}\left(G_{ps}\left(p_{i}\right)\right)\right)\right\|}_{2}^{2}\right)$$

where *j* represents the *j*th layer of the network, and *N_j_* represents the number of perceptrons in the *j*th layer. Simultaneously, Φ*_j_*(*x*) represents the feature map obtained by inputting the image *x* into the *j*th layer of the pre-trained VGG-19 network. In addition, due to the lack of local constraints, the generated face images often lack realistic details. In particular, mottle will occur in important facial areas such as the eyes and mouth of the generated image. To make the generated face photos retain more identity information, we define the facial detail feature loss from a regional perspective. According to the facial coordinate information, the regions around the eyes, nose, and mouth of the source image and the reconstructed image are segmented. As shown in Figure 6, the residual network Resnet50 is used to extract the features of several face regions of the source domain image and the reconstructed image. Then, the cosine distance of the corresponding regions of the two domain images is calculated. The final facial detail feature loss is obtained by adding the cosine distances of several regional features to constrain the generated image retain more details. The facial detail feature loss function is shown in Equation (12):

(12) $$l_{cyc\_face}=\overset{4}{\underset{c=1}{\sum }}Cos\left(\Phi \left(I_{c}^{ori}\right),\Phi \left(I_{c}^{cyc}\right)\right)$$

In Equation (12), *I^ori^* is the source domain image, *I^cyc^* refers the reconstructed image. Meanwhile, *I*_1_, *I*_2_, *I*_3_, and *I*_4_ represent the four image areas of the left eye, right eye, nose, and mouth of the image, respectively. Φ(*) represents the feature vector extracted by the Resnet50, and Cos represents the cosine distance. Therefore, the multi-level cycle consistency loss is expressed as Equation (13):

(13) $$l_{cyc}=\lambda_{1}L_{cyc\_pix}+\lambda_{2}L_{cyc\_feature}+\lambda_{3}L_{cyc\_face}$$

In summary, the total loss function is expressed as Equation (14), where *λ*_1_, *λ*_2_, *λ*_3_, *λ*_4_ are hyperparameters that control the importance of each part of the loss function.

(14) $$l_{total}=L_{LSGAN_{p}}+L_{LSGAN_{s}}+L_{cyc}+\lambda_{4}L_{Identity}$$

## 4. Results

This section begins with the details of the experimental settings. Experimental validation is performed on two public face sketch datasets: CUFS [3] and CUFSF [25]. Our method is compared with the six other models, namely Pix2Pix [20], CycleGAN [15], PS2MAN [8], CA-GAN [13], DivCo [26], and MSPC [27] in terms of visual presentation and evaluation indicators. We also add ablation experiments to verify the effectiveness of the proposed model.

### 4.1. Datasets

The CUFS dataset consists of three data subsets: CUHK, AR [28], and XM2VATS [29]. For each face, there exists a real photo and a corresponding sketch in the CUHK, AR, and XM2VATS datasets. The sample sizes of the three datasets of CUHK, AR and XM2VATS are 188, 123, and 295, respectively. The CUFSF database includes 1194 exaggerated face sketches, which correspond one by one to the gray real face photo in the FERET [30] dataset. Before the experiment starts, this paper divides training-testing set of different datasets according to Table 1.

### 4.2. Implementation Details

Our experiments are performed in PyTorch on an NVIDIA Quadro P4000 GPU. Before the model training process, the input sketch is aligned with key points according to the key position information of the face. The size of the input image is adjusted to 256 × 256. Finally, the synthesized photos are cropped from the size of 256 × 256 to the size of 250 × 200. The number of iterations of the model is 200 epochs in total. The learning rate for the first 100 epochs is set to 0.0002, and linearly decayed down to 0 for the last 100 epochs. The Adam optimizer with momentum parameters *β*_1_ = 0.5 and *β*_2_ = 0.999 is used to optimize the model. The hyperparameters in the objective function are set to *λ*_1_ = 10, *λ*_2_ = 10, *λ*_3_ = 10^−1^, *λ*_4_ = 5. To verify the effectiveness of our method, it compared the visualization results of each model. Next, Structural Similarity (SSIM) [31] and Peak Signal-to-Noise Ratio (PSNR) were used to evaluate the similarity between generated photos and real photos.

### 4.3. Comparative Analysis with Other Methods

#### 4.3.1. Qualitative Analysis

**Figure 7:**
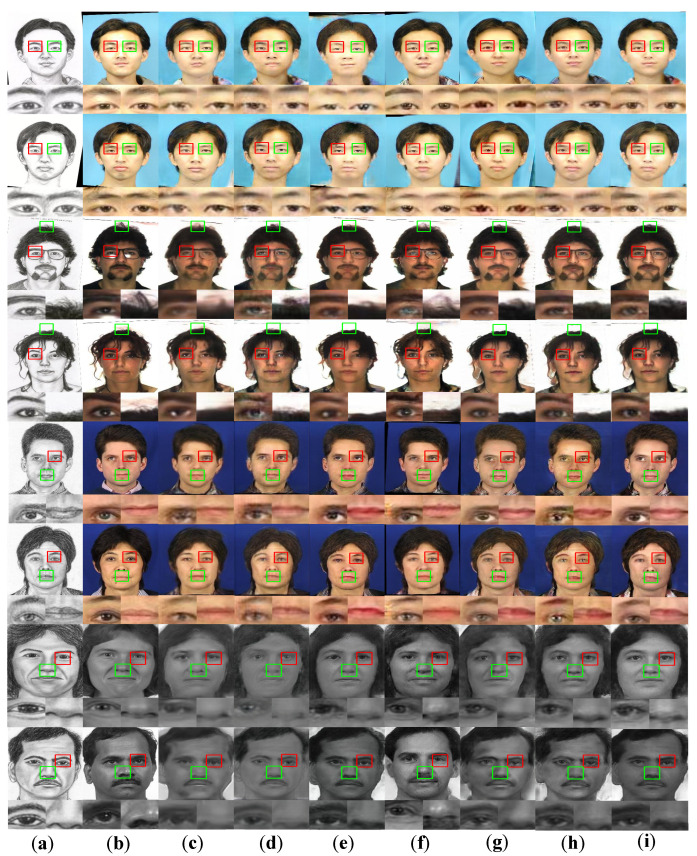
Comparison of synthetic results of different methods. The red and green boxes clearly show the details of the photos synthesized by different methods. (**a**) Original sketch; (**b**) Real Photo; (**c**) Pix2Pix; (**d**) CycleGAN; (**e**) PS^2^MAN; (**f**) CA-GAN; (**g**) DivCo; (**h**) MSPC; (**i**) Ours.

Figure 7 recorded the synthetic results of our method and other methods on data from CUFS dataset and the CUFSF dataset. The first 6 rows of Figure 7 were the experimental results of various methods on the CUFS dataset. It was found that the CycleGAN method could obtain complete facial components. However, photos synthesized by CycleGAN have low-resolution and gave a hazy feeling. There were noise and artifacts in the local area of face photos synthesized by Pix2Pix method. The above two methods were only constrained at the pixel level and could not synthesize photos that were clearer and contained key facial details. The experimental results obtained by the PS2MAN method had higher resolution, but also lacked some face details. The CA-GAN method produced sharper photos with fewer noise artefacts than the above method, which was closer to the real photo. The photos synthesized by the DivCo method successfully retained most of the facial details, but some of the synthesized face photos would be affected by the background color and produced abnormal color blocks. The photos synthesized by the MSPC method had noise blobs in key detail areas such as the eyes. Compared with our method, the skin color of the face image generated by CA-GAN was closer to the real photo, but our method did better in the key details and edge structure of the face.

The last two rows of Figure 7 showed the synthetic results on the CUFSF dataset. Compared with the CUFS dataset, the lines of the face sketch in the CUFSF dataset were too rough. Moreover, the characters in the CUFSF dataset did not belong to the same race, and their appearances were quite different. Therefore, the synthesis task on the CUFSF dataset was extremely challenging. The synthetic results obtained by the Pix2Pix and the CycleGAN method had a large number of mottled artifacts. The translation results of the PS2MAN method reduced the artifacts, but the synthetic results lacked key facial details. The face synthesized by the DivCO method on the CUFSF dataset was not realistic enough, and the skin color was between the sketch and the photo. The edge structure of the image synthesized by MSPC method had slight local blurring. Compared with the above methods, the synthesis results of CA-GAN and our method greatly retained more key details and were closer to real photos.

#### 4.3.2. Quantitative Analysis

The SSIM and the PSNR were used as evaluation metrics to evaluate the experimental results. Among them, the SSIM was an index used to compare the difference of structural information between synthetic images and real images, which was more in line with human visual perception. The PSNR was a measure of whether the synthetic image was close to the real image at the pixel level. Therefore, these two evaluation indicators could estimate the quality of synthesized images of different methods.

As shown in Table 2 and Table 3, this paper compared the evaluation indicators obtained by the proposed method with other methods on various public datasets. Table 2 and Table 3 showed that our method achieved the highest SSIM value on AR and XM2VTS datasets, highest PSNR value on AR, XM2VTS, and CUFSF datasets. Although the scores of the CA-GAN model were higher than our method on the CUHK dataset, it could be seen that the gap was not large. The data in Table 2 and Table 3 were basically consistent with the visual results shown in Figure 7. From the data in the table, it could be concluded that the evaluation index of our method on public datasets was basically better than other methods. The value of SSIM can also be proved that the face photos synthesized by our method were closer to real face photos.

### 4.4. Ablation Experiment

**Figure 8:**
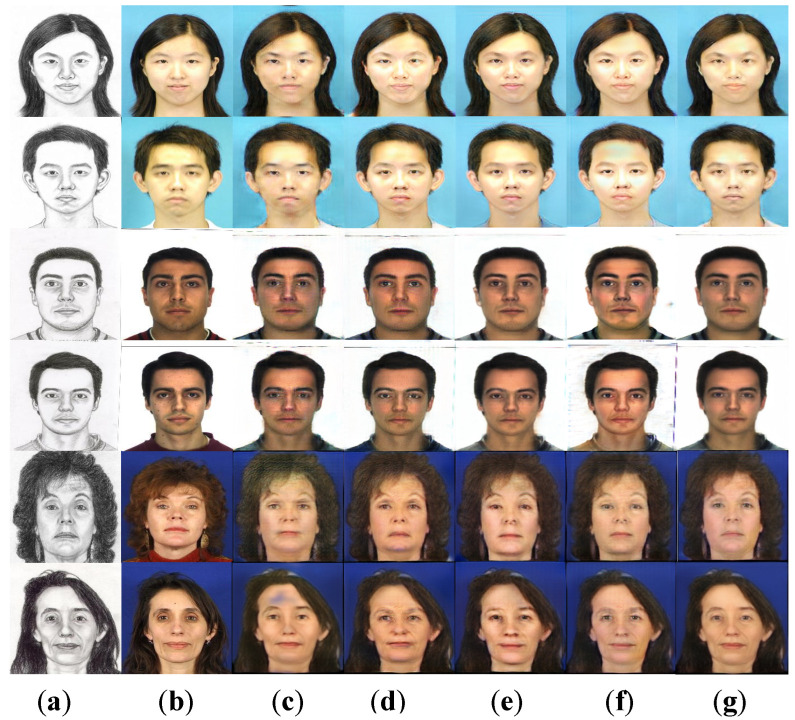
Comparison of synthetic results of ablation experiment: (**a**) Original sketch; (**b**) Real Photo; (**c**) CycleGAN; (**d**) CycleGAN w/MFEM; (**e**) CycleGAN w/CBAM-R; (**f**) CycleGAN w/Multi-level cycle loss; (**g**) Ours.

To further demonstrate the effectiveness of each module in our method, ablation experiments were performed on the CUFS dataset. Using CycleGAN as the base network, several different model variants were built. As shown in Table 4 and Figure 8, our model was compared with three combinations of CycleGAN w/ MFEM, CycleGAN w/ CBAM-R, and CycleGAN w/ Multi-level cycle loss for quantitative and qualitative experimental comparisons. “w/” means with.

According to the experimental indicators in Table 4, it can be seen that the introduction of the three improved parts could obtain higher SSIM and PSNR values than the baseline, and thus we could obtain synthetic results closer to the real face. The first and second columns in Figure 8 were the input sketches and corresponding photos. It can be seen that there were some black artifacts in the face photos synthesized by the CycleGAN model in the third column, and the key parts of the face such as the eyes and nose were relatively blurred. As shown in Figure 8d,e, adding a multi-scale extraction module and an attention residual block increases the model’s ability to extract feature information. The synthesized face photos reduced artifacts and noise. However, due to the lack of local constraints, the facial details were too smooth and lost the original texture details. As shown in Figure 8f, the model improved the ability to constrain facial details by applying a multi-level cycle consistency loss. However, the synthetic photos were slightly different from real photos in terms of realism. As shown in Figure 8g, the face photos synthesized by our model were more realistic than the previous basic network, and the edge structure and facial details were clearer and more complete.

## 5. Conclusions and Future Work

In this paper, we proposed an unsupervised CycleGAN-based model for converting face sketches into high-quality photos. A multi-scale feature extraction module was designed in front of the generator to enable the model to extract multi-scale feature information. Meanwhile, we introduced CBAM within the network to adjust the weight of important feature information more accurately and reduce the interference of irrelevant factors, such as background. The introduced perceptual loss and facial detail feature loss reduced the loss of information in the transformation process and preserved more facial structural information. Compared with previous models, the model proposed in this paper did not have the limitation of requiring paired data. Regardless of subjective visual effects or objective evaluation indicators, compared with other models, the quality of the synthesized images on the CUFS and CUFSF public datasets had been improved. Moreover, the synthesized photos had more complete details and clearer edge structures. The model in this paper was only suitable for frontal face image synthesis, and thus a subsequent work will consider heterogeneous face synthesis from multiple perspectives.

## Figures and Tables

**Table 1:** Partition of dataset.

Dataset	CUHK	AR	XM2VATS	CUFSF
Training numbers	100	80	100	200
Testing numbers	88	43	195	994

**Table 2:** SSIM comparison of photos synthesized by different models on the data.

Methods	CUHK	AR	XM2VTS	CUFSF
Pix2Pix	0.647	0.676	0.548	0.529
CycleGAN	0.631	0.657	0.556	0.548
PS^2^MAN	0.653	0.689	0.562	0.583
CA-GAN	0.702	0.693	0.587	0.613
DivCo	0.657	0.635	0.590	0.547
MSPC	0.679	0.647	0.562	0.556
Ours	0.688	0.714	0.612	0.607

**Table 3:** PSNR comparison of photos synthesized by different models on the data.

Methods	CUHK	AR	XM2VTS	CUFSF
Pix2Pix	16.588	17.212	18.233	16.436
CycleGAN	16.356	16.835	18.161	16.386
PS^2^MAN	17.688	17.032	18.483	16.695
CA-GAN	18.472	17.431	18.605	17.120
DivCo	16.836	17.223	18.602	16.431
MSPC	17.771	17.353	18.414	16.785
Ours	17.896	17.683	18.892	17.358

**Table 4:** Comparison of experimental indexes under ablation experiment.

Methods	CUHK		AR		XM2VTS	
	SSIM	PSNR	SSIM	PSNR	SSIM	PSNR
CycleGAN	0.631	16.356	0.657	16.835	0.556	18.161
CycleGAN w/MEFM	0.647	16.751	0.679	17.235	0.568	18.327
CycleGAN w/CBAM-R	0.658	17.563	0.683	17.371	0.599	18.796
CycleGAN w/Multilevel cycle loss	0.663	17.458	0.696	16.897	0.593	18.463
Ours	0.688	17.896	0.714	17.683	0.612	18.892

## Data Availability

The code supporting the conclusions of this article will be made available by the corresponding author upon reasonable request.
